# Hypothalamic Expression of Melanocortin-4 Receptor and Agouti-related Peptide mRNAs During the Estrous Cycle of Rats

**Published:** 2014

**Authors:** Mohammad Reza Zandi, Mohammad Reza Jafarzadeh Shirazi, Amin Tamadon, Amir Akhlaghi, Mohammad Saied Salehi, Ali Niazi, Ali Moghadam

**Affiliations:** 1 Department of Animal Sciences, School of Agriculture, Shiraz University, Shiraz, Iran.; 2 Transgenic Technology Research Center, Shiraz University of Medical Sciences, Shiraz, Iran.; 3 Biotechnology Research Center, School of Agriculture, Shiraz University, Shiraz, Iran.

**Keywords:** Melanocortin- 4 receptor, agouti- related peptide, hypothalamus, estrous cycle, rat

## Abstract

Melanocortin- 4 receptor (MC4R) and agouti- related peptide (AgRP) are involved in energy homeostasis in rats. According to MC4R and AgRP effects on luteinizing hormone (LH) secretion, they may influence the estrous cycle of rats. Therefore, the aim of this study was to investigate the expression of MC4R and AgRP mRNAs at different stages of estrous cycle in the rat’s hypothalamus. The estrous cycle stages (proestrus, estrus, metestrus and diestrus) were determined in 20 adult female rats using vaginal smears. The rats were divided into four equal groups (n=5). Four ovariectomized rats were selected as controls two weeks after surgery. Using real- time PCR, relative expressions (compared to controls) of MC4R and AgRP mRNAs in the hypothalamus of rats were compared in four different groups of estrous cycle. The relative expression of MC4R mRNA in the hypothalamus of female rats during proestrus stage was higher than those in other stages (P=0.001). Despite a lower mean of relative expression of AgRP mRNA at proestrus stage, the relative expression of AgRP mRNA of the four stages of estrous cycle did not differ (P>0.05). In conclusion, changes in the relative expression of MC4R and AgRP mRNAs in four stages of rat estrous cycle indicated a stimulatory role of MC4R in the proestrus and preovulatory stages and an inhibitory role of AgRP in gonadotropin releasing hormone (GnRH) and LH secretions.

Melanocortin-4 receptor (MC4R) is the cognate receptor for α- melanocyte stimulating hormone (α-MSH) (1). This receptor belongs to the melanocotrin receptors and is one of the members of G protein-coupled receptors that activates adenylate cyclase and can be a mediator between appetite and reproduction (2). MC4R is expressed in arcuate nuclei (ARC), periventricular nuclei (PVN), medial preoptic area (MPO) and preoptic area (POA). These regions are involved in reproduction and regulate appetite (3, 4). Alpha-melanocyte stimulating hormone is effective in erection (5) and decreases luteinizing hormone (LH) in rats (6). Melanotan-II (MT-II) -agonist of MC4R- is effective in the erection of men and treatment of its abnormalities (7).

Agouti-related peptide (AgRP) is the neuropeptide with 132 amino acids that is expressed in the ARC of mice (8) and sheep (9). In some species, AgRP neurons are involved in energy balance (10). AgRP is an appetite stimulus (11) and increases during the lack of energy (12). Intracerebroventricular (ICV) injection of AgRP, increases food intake and inhibits α-MSH in mice (13). Mice deficient in the MC4R expression (14) or high expression of AgRP (15) are fat. Changes in the relative expression of MC4R and AgRP mRNAs during long term malnutrition of rat indicate a stimulatory role of MC4R and AgRP in regulating energy balance in ARC of rat hypothalamus (16). Various hormones such as insulin, leptin and glucocorticoids can change AgRP expression during homeostasis (11). Moreover, AgRP in pregnancy and MC4R in lactation in ARC of rats control the energy homeostasis (17).

Many neurons in the ARC co-express AgRP and Neuropeptide Y (NPY) which is another appetizer peptide (18). NPY is also effective in reproduction (19). It has been shown that NPY neurons have synaptic contact with gonadotropin releasing hormone (GnRH) neurons in the POA (20). ICV injection of NPY, inhibits LH secretion in rats (21). Moreover, NPY neuron termini which synthesized AgRP, synapse with GnRH neurons (22). Both NPY and AgRP inhibit LH surge (3, 23). Estradiol decreases NPY and AgRP expression in the ARC as well as NPY expression in the PVN of ovariectomized (OVX) rats (24). In addition, the low level of estradiol in OVX rat, leads to the up-regulation of NPY and AgRP in the hypothalamus (25). AgRP termini are located in the MPO which consists of GnRH neurons and ICV injection of AgRP increases GnRH, LH, follicle stimulating hormone (FSH) and testosterone in male rat but do not have any direct effect on anterior pituitary (26). AgRP inhibitor also has effect on reproductive axis and ICV injection of AgRP inhibitor leading to LH decline in OVX monkey (23).

According to the effect of MC4R and AgRP on LH secretion, there are likely to affect estrous cycle in rats. Therefore, the aim of the present study was to investigate MC4R and AgRP mRNAs expression in the hypothalamus during the estrous cycle of rat.

## Materials and Methods

Twenty-four adult (3-4 months old) female Sprague-Dawley rats (*Rattus norvegicus*) weighing 170-220 g were used in this study. The rats were randomly selected and housed in the laboratory animal center of Shiraz University of Medical Sciences, Shiraz, Iran under controlled temperature (22 °C) and light (12:12 light to dark ratio; lights on at 7:30 am) conditions. Rats were treated humanely and in compliance with the recommendations of the Animal Care Committee of the Shiraz University of Medical Sciences. All experimental procedures were carried out between 12.00-2.00 pm. Vaginal smears were prepared for the identification of the phases of the estrous cycles of the 20 intact rats. Five rats were assigned to each phase of the cycle.

The control group comprised four randomly selected ovariectomized rats. The rats were anesthetized by an intraperitoneal injection of ketamine (100 mg/kg, Woerden, Netherlands) and xylazine (7 mg/kg, Alfazyne, Woerden, Nether-lands), then ovariectomized through a ventral midline incision. Further procedures were under-taken after a two-week recovery period. The cyclic and ovariectomized rats were decapitated, brains dissected out immediately, and the entire hypo-thalamuses were dissected. The hypothalamus samples were frozen in liquid nitrogen and stored at-80 °C. RNA extraction, DNase treatment, cDNA synthesis and relative real-time PCR procedure were performed as described elsewhere (27). Primers were designed with Allele ID 7 software for the reference gene, AgRP (NM_033650.1) and MC4R (NM_013099.2). The rat glyceraldehyde-3- phosphate dehydrogenase (GAPDH) gene (M32599) was used as reference gene for data normalization (Table 1).

For quantitative real-time PCR data, the relative expression of AgRP and MC4R was calculated based on the threshold cycle (CT) method. CT for each sample was calculated using Line-gene K software (28). Fold expression of the target mRNAs over reference values was calculated by the equation 2-ΔΔCT (29), where ΔCT is determined by subtracting the corresponding GAPDH CT value (internal control) from the specific CT of the target (AgRP or MC4R). ΔΔCT was obtained by subtracting the ΔCT of each experimental sample from that of the calibrator sample (ovariectomized rats). Data on the relative expression of AgRP and MC4R genes were subjected to the test of normality. Analysis of variance for both variables were performed using Proc GLM (SAS, 2002) followed by mean comparison by Duncan`s multiple range test. P<0.05 was considered as significant. The mean of the group and standard errors have been reported.

## Results

**Fig. 1:**
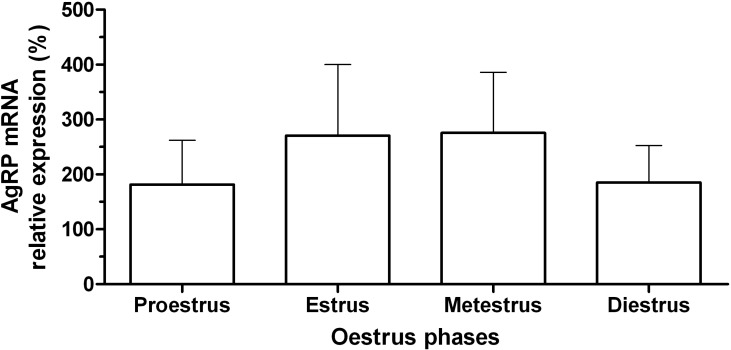
Mean (± standard error) of the relative expression of MC4R gene in the hypothalamus of rats (n=5) during the estrous cycle. Different letters indicate significant difference (P<0.05).

**Fig. 2:**
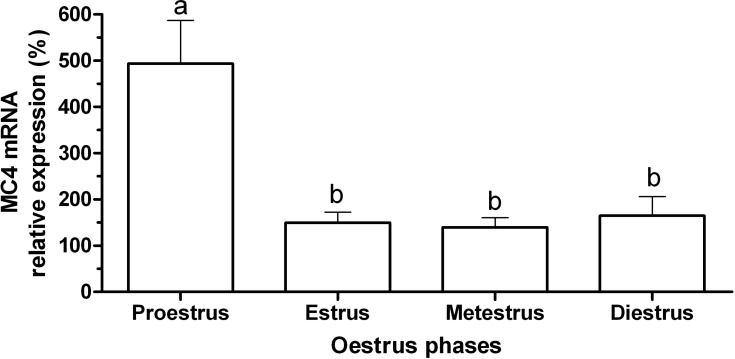
Mean (± standard error) of the relative expression of AgRP gene in the hypothalamus of rats (n=5) during the estrous cycle

The expression of MC4R mRNA in the hypothalamus of female rats at different phases of the estrous cycle is shown in figure 1. There was higher expression of MC4R mRNA during the proestrus phase compared to other phases of the cycle (P=0.001). The expression of MC4R mRNA during the estrus, metestrus and diestrus phases did not differ significantly. The expression of AgRP mRNA in the hypothalamus of female rats in the different phases of the estrous cycle is shown in figure 2. The expression of AgRP mRNA did not significantly differ during the estrous cycle (P>0.05). Negative correlations between AgRP mRNA and MC4R mRNA during the estrous cycle were not significantly different (r= -0.14, P> 0.05).

**Table 1:** Sequences of PCR primers used to evaluate relative expression of AgRP and MC4R genes in rat

**Amplicon length (bp)**	**Primer sequence**	**Primer**
181	5` TGGGTGTCATAAGCCTGTTGG 3` 5` GCGTCCGTGTCCGTACTG 3`	MC4R-F MC4R-R
189	5` TGAAGAAGACAGCAGCAGACC 3` 5` TGAAGAAGCGGCAGTAGCAC 3`	AgRP-F AgRP-R
112	5` AAGAAGGTGGTGAAGCAGGCATC 3` 5` CGAAGGTGGAAGAGTGGGAGTTG 3`	GAPDH-F GAPDH-R

## Discussion

In the present study, we investigated the expression of AgRP and MC4R mRNAs during the estrous cycle in the rat hypothalamus for the first time. We also showed that MC4R mRNA in proestrus was greater (3.2 fold) than the other phases of the cycle. In rats during the estrus, metestrus, diestrus, and early proestrus phases, the concentration of GnRH is at its basal level. At mid-proestrus phase, the GnRH surge center is activated (30). In the afternoon of proestrus phase, the circulating levels of LH begin to rise rapidly and reaches its peak in the evening, resulting in ovulation. The blood LH level then decreases and reaches basal levels during the rest of the cycle (31). Therefore, increase in MC4R mRNA that we observed in the present study might be involved in preovulatory GnRH/ LH surge. Most of the GnRH neurons are located in the vicinity of α-MSH expressing fiber (32) and GT1-1 hypothalamic cells express MC4R and the stimulation of these cells with Nle4 D-Phe7-α-MSH leads to GnRH secretion (33, 34).

In rats, ovarian estradiol secretion reaches its maximum level in the mid of the proestrus phase (31). Estradiol injection led to the up-regulation of MC4R in the PVN of OVX rats (35). Ovariec-tomized rats that had been treated with estrogen and progesterone indicated partial prolactin and LH surge that was inhibited during hunger (36). MC4R antagonists decrease prolactin and LH surge in the rats fed with normal diet and inhibit the effect of leptin on these hormones in hungry rats. Thus, MC4R could be a mediator of leptin effects on prolactin and LH surge (36). MT-II led to prolactin surge in hungry rats. Accordingly, MC4R may be important for prolactin surge during the preovulatory period (37). Leptin stimulates GnRH (38) and LH secretion in the rat hypothalamus and MC4R is a mediator of leptin effects during homeostasis (39). Therefore, the high level of MC4R mRNA during the proestrus phase could be involved in prolactin preovulatory surge.

In the present study, there was no significant difference in AgRP mRNA levels during the estrous cycle but the mean of AgRP mRNA in proestrus phase was 40% lower than estrus and metestrus phases and 10% lower than diestrus phase. In many mammals, GnRH secretion is regulated by ovarian steroid feedback mechanisms. In rats, ovarian estradiol secretion during the estrus phase is low, while at the end of metestrus phase, estrogen secretion begins to increase and is high during the diestrus phase; it reaches its peak in the proestrus evening, thereafter, declining to its basal level (31). In OVX rats, estradiol injection led to the decrease of AgRP expression in the ARC (25). Therefore, the low level of AgRP mRNA in proestrus and diestrus phases could be due to the increase of estradiol secretion during these phases.

In rodents, GnRH neurons do not express alpha- estradiol receptor (ERα) that is essential for positive and negative estradiol feedback (40). Increase in tonic LH secretion leads to the increase in estradiol synthesis from follicles and the peak of estradiol leads to LH surge (41). AgRP inhibits prolactin and LH surge in female rats while the antiserum against AgRP reverses this effect (42). ICV injection of AgRP, inhibits pulsatile LH secretion in monkey (23). Thus, AgRP could inhibit GnRH pulses and regulate estrous cycle.

In conclusion, our finding showed that in proestrus phase during the preovulatory period, MC4R might have excitatory effects and AgRP might have inhibitory effects on GnRH/ LH secretion in rats.
